# Swimming exercise is a promising early intervention for autism‐like behavior in *Shank3* deletion rats

**DOI:** 10.1111/cns.13920

**Published:** 2022-10-11

**Authors:** Dan Xu, Yunchen Meng, Shasha An, Wenshu Meng, Hanran Li, Weinan Zhang, Yaqi Xue, Xinyu Lan, Xiaoxi Wang, Mingjuan Li, Xiaoyan Zhang, Zhang Zhihao, Yu Zhao, Haodong Yang, Chen Zhang, Rong Zhang, Zhiping Zhen

**Affiliations:** ^1^ College of P.E and Sports Beijing Normal University Beijing China; ^2^ Sports and Health Editorial Office People's education press Beijing China; ^3^ Department of P.E. China University of Mining and Technology‐Beijing Beijing China; ^4^ College of Life Sciences Beijing Normal University Beijing China; ^5^ Centre for Cognitive and Brain Sciences and Department of Psychology University of Macau Taipa Macau; ^6^ Department of Neurobiology, School of Basic Medical Sciences Peking University Beijing China; ^7^ Neuroscience Research Institute Peking University Beijing China; ^8^ Key Laboratory for Neuroscience, Ministry of Education/National Health and Family Planning Commission Peking University Beijing China; ^9^ Autism Research Center of Peking University Health Science Center Beijing China; ^10^ Institute of Acupuncture and Moxibustion China Academy of Chinese Medical Sciences Beijing China; ^11^ Department of Neurobiology, School of Basic Medical Sciences, Beijing Key Laboratory of Neural Regeneration and Repair, Advanced Innovation Center for Human Brain Protection Capital Medical University Beijing China; ^12^ Department of Integration of Chinese and Western Medicine School of Basic Medical Sciences, Peking University Beijing China

**Keywords:** autism, early intervention, hippocampus, *Shank3*, swimming exercise

## Abstract

**Introduction:**

SHANK3 is an important excitatory postsynaptic scaffold protein, and its mutations lead to genetic cause of neurodevelopmental diseases including autism spectrum disorders (ASD), Philan McDermid syndrome (PMS), and intellectual disability (ID). Early prevention and treatment are important for *Shank3* gene mutation disease. Swimming has been proven to have a positive effect on neurodegenerative diseases.

**Methods:**

*Shank3* gene exon 11–21 knockout rats were intervened by a 40 min/day, 5 day/week for 8‐week protocol. After the intervention, the rats were tested to behavioral measures such as learning and memory, and the volume and H‐spectrum of the brain were measured using MRI; hippocampal dendritic spines were measured using Golgi staining and laser confocal.

**Results:**

The results showed that *Shank3*‐deficient rats had significant deficits in social memory, object recognition, and water maze learning decreased hippocampal volume and number of neurons, and lower levels of related scaffold proteins and receptor proteins were found in *Shank3*‐deficient rats.

**Conclusion:**

It is suggested that early swimming exercise has a positive effect on *Shank3* gene‐deficient rats, which provides a new therapeutic strategy for the prevention and recovery of neurodevelopmental disorders.

## Graphical abstract

Eight weeks of swimming training make *Shank3* knockout pups improve autistic‐like behaviors such as learning memory in rats, increase hippocampal volume, and increase dendritic spine complexity in hippocampal neurons.

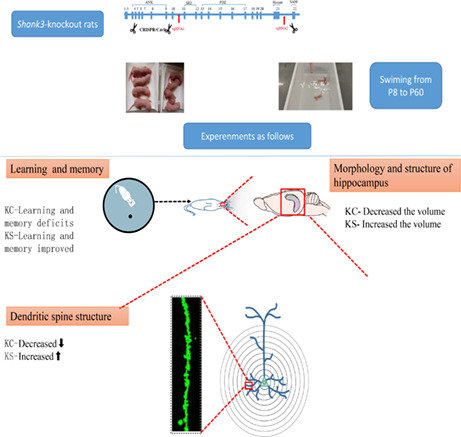

## 1 INTRODUCTION

Autism spectrum disorder (ASD) is a neurodevelopmental disorder that affects one in 44 children in the United States.^1^ Individuals diagnosed with ASD show impairment in social interaction and communication and restrictive, repetitive behaviors from an early age.^2^ In the last two decades, the etiology of ASD has been shown to be extremely complex, involving both genetic and epigenetic variations and environmental influences.^3^, ^4^ These risk factors may lead to abnormal development of synapses or brain circuits.^5^ Recent genetic screening results have shown that, in addition to being involved in PMS, *Shank3* mutations are also associated with nonsyndromic ASD.^6^, ^7^ Nowadays, drug therapy is used to target common comorbidity behaviors such as anxiety, hyperactivity, and impulsivity in autistic individuals.^8^, ^9^, ^10^ However, we are still facing greater challenge in effective treatment on the core autistic behaviors, including social–communicational difficulties and repetitive/restricted interests.

SHANK3 is a member of the SHANK1‐3 protein family and it interacts with many postsynaptic density (PSD) proteins.^11^ Positive and negative NMDAR modulators have been shown to alleviate ASD symptoms in patients and normalize ASD‐like phenotypes in animal models.^12^ *Shank3* is a recognized autism risk gene, and detailed mechanismic research provides insight into its role in drosophila, mice, rats, monkeys, and other species.^2^, ^13^, ^14^, ^15^, ^16^, ^17^ Compared with mice, the rat model has species‐specific advantages and is a more typical pharmacological model with a wider behavioral paradigm, able to perform more complex social tests and voice ultrasonic tests and exhibiting a more established brain region division. Thus, the construction of a autism rat model with a large fragment deletion of *Shank3* may provide further information about the pathogenesis of ASD and allow for the exploration of the mechanisms of various behavioral and motor characteristics.

Early life experience may change the brain network and affect adolescents' emotions and cognition.^18^ Similarly, for brain diseases, early diagnosis and intervention can also produce a better prognosis.^19^, ^20^ In recent years, researchers have reached a consensus that early detection and intervention can optimize the prognosis of children with ASD.^21^, ^22^, ^23^ Swimming can improve cognitive and mental health.^24^, ^25^ Research shows that water sports are effective in improving ASD children's behavior, emotions, and social and motor skills.^26^, ^27^, ^28^ After long‐term regular swimming exercise, ASD patients' water sports skills and spontaneous social interaction behavior increased significantly,^29^ their water safety skills were dramatically improved,^30^ and their frequency of stereotyped behavior was apparently reduced.^31^ Therefore, the effect of early swimming should be given more attention.

## 2 METHODS AND MATERIALS

### 2.1 Experimental animals and grouping

All the rat models used in this experiment were male *Shank3* knockout homozygous rats constructed in cooperation with the Neuroscience Research Institute, Peking University. SD rats were used to perform exon knockout of segments 11–21 at the embryonic stage using CRISPR/Cas9 Technology. Altered behaviors and impaired synaptic function were observed in the novel rat model with complete *Shank3* deletion.^13^

Genotypes were determined by PCR of rat tail DNA using the primers F1 (CTGTTGGCTGAGCCTGGCATAGAG) and R1 (GCTGGAAAGAAACAACGAGAGCCAG) for the WT allele (559 base pairs) and the primers F2 (TTGTGCACTGCCTATGTTGACCACT) and R2 (TAGGCGAGAGAAGATGGTGTGATTTCC) for the mutant allele (688 base pairs) (Figure S1A).

For analysis of SHANK3 protein expression, Western blots were performed. The results showed that no SHANK3 isoform was absent in the hippocampal brain region of *Shank3* knockout rats (Figure S1B).

Forty‐four *Shank3* knockout homozygous and littermate wild‐type rats were divided into four groups: wild‐type control group (WC, *n* = 9), wild‐type swimming (WS, *n* = 11), *Shank3* control group (KC, *n* = 10), and *Shank3* swimming group (KS, *n* = 14). All animals were housed with free access to a standard laboratory diet and water with a 12‐h light–dark cycle under standard conditions (indoor temperature 22 ± 1°C, humidity 65%–70%). This study was carried out following the USA National Institutes of Health Guide for the Care and Use of Laboratory Animals. The protocol was approved by the Peking University Animal Care and Use Committee (ethics approval ID, LA2015204).

### 2.2 Swimming exercise protocol

When the pups reached 8 days of age, early swimming training was performed for 8 weeks with pups of the KS and WS groups. The age of 8 days corresponds to 1 year for young children.^32^ In this study, a well‐established early swimming protocol for 8‐day‐old SD rats was used.^33^ The young rats underwent adaptive self‐training for the first 2 weeks, which was gradually increased to 40 min/session from 2 min/session five times a week. Beginning at Week 3, the young rats underwent swimming training of 5 cycles/week, 40 min/session, and self‐weighted until 8 weeks. The training period was performed on Monday to Friday afternoons, and they were rested on weekends. The detailed protocol was:

8 days old, swimming for 2 min, water temperature of 32°C, water level was the height of the legs.
9 days old, swimming for 5 min, the water level was the height of the stomach.
10 days old, swimming for 10 min., the water level was the height of the neck.
2 days old, rest.
13–17 days old, swimming at 15, 20, 25, 30, and 40 min daily with a normal swimming water level.
20–36 days old, swimming for 40 min daily, swimming for 5 days, and resting for 2 days.
36–64 days old, swimming for 40 min daily. Sixty‐four days old was the end day of the 8‐week intervention.

### 2.3 Behavior tests

#### 2.3.1 Three‐chamber test

*Experimental setup*: A 40 × 34 × 24 cm Plexiglas box was divided into three chambers, A, B, and C, and the corridor size was 10 cm × 10 cm × 15 cm. Chambers A and B were located at both ends of the box, chamber C was in the middle, and the activity of the rats in the three chambers was automatically monitored by a computer. During the acclimation phase, the tested rats were allowed to explore the entire apparatus freely for 5 min. During phase 1, the social preference test phase, the tested rats were removed from the three chambers. Model rat A (sex‐ and age‐matched wild‐type model rat) was placed in a metal cage in the A side box, and this was social stimulus 1 (Model A); an empty metal cage was placed in the B side box as a nonsocial stimulus (empty). Subsequently, the test rats were placed in the middle box and allowed to explore freely for 10 min. During phase 2, Model rat A was still placed ipsilateral to stage 1, model rat B was placed on the empty cage side of stage 1 (Model B), and the experimental rat was placed in the middle zone and recorded for 10 min. The floor plate and standing plate were wiped clean using 75% alcohol after the end of each stage. Statistics were performed by 2 fellow students blinded to the genotype, timed back‐to‐back double‐blind, and averaged for the calculation.

#### 2.3.2 Morris water maze test

One day before the experiment, the rats were pretested for familiarity with the water, and each rat swam freely in the pool for 2 min. In the experiments, the rats were trained eight times per day using stainless steel cylindrical buckets (150 cm diameter, 50 cm height) for 5 days. One of the 4 quadrants was selected as an entry point each time. The rat was gently placed into the water, and the time from entering the water to climbing onto the platform was recorded (escape latency). Spatial search experiments explored the ability of the rats to accurately recall the spatial location of the original platform. On Day 6 of the experiment, the platform was removed, and the rats were gently placed at an arbitrary entry point in the water. The spatial search time of the rats was 90 s, and the time the rats spent in the target quadrant (the original platform quadrant) and the other quadrants for 90 s was calculated. If the rat could not find the platform within 90 s, the rat was placed on the platform and allowed to stay there for 30 s.

#### 2.3.3 Novel object recognition test

Learning and memory were assessed by a new object recognition test, which was lit up in dark red, with some modifications, as in previous studies.^34^ During the acclimation phase, the rat was placed into the test box (60 × 60 cm) for 10 min. During the study phase, 2 identical objects were placed in the test box, and the rats were placed into the test box and allowed to explore for 20 min. During the learning phase, which required the placement of 2 identical objects in the test box, the rats were placed in the test box and allowed to freely explore for 20 min. One hour after the learning phase, one of the 2 identical objects in the test box was replaced with another object that was similar in volume, shape, and color (the novel object). The test rat was again placed into the test box, allowed to explore freely for 10 min, and videotaped. After each stage of the experiment, the test box and objects were wiped with 75% alcohol to eliminate the interference of residual odor in subsequent experiments. Statistics of the sniffing time of the tested rats toward both objects in the test phase were defined as the sniffing behavior when the rat's nose tip was facing toward the object and when the distance from the object was within 2 cm. Statistics were performed by 2 fellow students blinded to the genotype, timed back‐to‐back double‐blind, and averaged for calculation.

### 2.4 MRI acquisition and analysis

Rats were anesthetized with isoflurane anesthesia before MRI scanning, and the vital signs of the rats were monitored. The sequence and parameters of MRI scanning with 7.0 Bruker were as follows. VBM set parameters, repetition time tr: 11400 MS, echo time TE: 48 ms, Rare factor: 8, NEX (number of average): 6, layer thickness: 0.35 mm, layer intercept: 0, layer number: 80, FOV: 35 × 35 mm, matrix: 256 × 256. DIT setting parameters: TR: 10,000 ms, TE: 30.40 ms, segment: 4, diffusion gradient duration: 4 ms, diffusion gradient separation: 15 ms, number of diffusion director: 30, number of B0 (A0) images: 5, B value per director: 1000 s/mm^2^, layer thickness: 0.7 mm, layer intercept: 0, layer number: 40, FOV: 35 × 35 mm, matrix: 128 × 128. H proton spectroscopy parameters, location: hippocampal CA1 brain area, slice 36–41 in VBM, volume: up and down 1.5 mm, left and right 2.5 mm, front and back 2 mm, tr: 2500 ms, Te: 20 ms, Na (repeat times): 512 times.

The preprocessing of the MRI data included five steps: time correction, motion correction, origin correction, space standardization, and smoothing. The VBM structure data were processed by spm8 software. First, all T2 images were extracted by the PCNN toolkit, and the brain tissue mask file was generated. The SPM image calculator was used to modify the extracted brain mask to obtain a better brain template. After smoothing, SPM was used for statistics to extract the voxel information. We used Xjview for visual analysis.

### 2.5 Golgi staining

According to the manufacturer's instructions, 9‐week‐old rats were subjected to Golgi's method with a Hito Gaz Christopher Cox Optimization Kit (HTKNS1125, HITOBIOTEC). In short, the brain was removed at room temperature in darkness and transferred to an immersion solution mixture containing solutions 1 and 2 of equal volume for 2 weeks. The brain was soaked at room temperature for 12–24 h in more than 5 volumes of solution, and then, the soaking solution was changed and it was stored for 14 days under the same conditions. After 14 days, the tissue became yellow–brown and was carefully moved to solution 3 (at least 5 times its volume). The tissue was stored in the dark at 4°C for 24–72 h, placed in a fresh volume of solution 3, and then stored in the dark at 4°C for 12 h.

Finally, sections with a thickness of 150 μm were cut at −19°C and then dried. The slides were stained with solutions 4 and 5. Finally, the stained sections were imaged using electron microscopy and laser scanning confocal microscopy (TCS‐SP8 STED 3X, Leica) with reflected light imaging; format: 512 × 512, ZOOM factor 0.75, large map Z set to 2, small map Z set to 0.3.

### 2.6 Analysis of morphological indices of the neurons

Electron microscopy was used to analyze the neuronal morphological indices. Three rats were selected from each group, and the clearest brain slice of the hippocampal CA1 region was selected for observation. Two neuronal dendrites were photographed under a 10× eyepiece (electron microscope) for each brain slice, and the neuronal complexity was analyzed using Sholl analysis. Dendritic spine density was analyzed using laser confocal microscopy by photographing 2 dendritic branches for each CA1 neuron under a 40× eyepiece. Neurons were subjected to Sholl analysis using ImageJ software, and individual dendritic spine densities were analyzed.

### 2.7 Statistics

SPSS 20.0 (SPSS Inc.) software was used for statistical analysis of all data. The results were expressed as mean ± SD. The results were plotted by GraphPad Prism 8 (GraphPad Software Inc.) software. The Shapiro–Wilk normality test was used to determine whether the data conformed to a normal distribution. For the comparisons, parametric tests including t‐tests and one‐way analysis of variance (ANOVA) were used if the data were normally distributed (distribution tested by the Shapiro–Wilk normality test), and nonparametric approaches, including the Wilcoxon test and Kruskal–Wallis test, were used for data with a nonnormal distribution. For all data, the results were expressed as the mean ± standard error of the mean (SEM), and *p* < 0.05 (two‐tailed) was considered statistically significant.

## 3 RESULTS

### 3.1 Swimming exercise rescues the social preference behavior, learning, and memory abilities of *Shank* 3 KO rats

#### 3.1.1 Social preference and social memory

**FIGURE 1:**
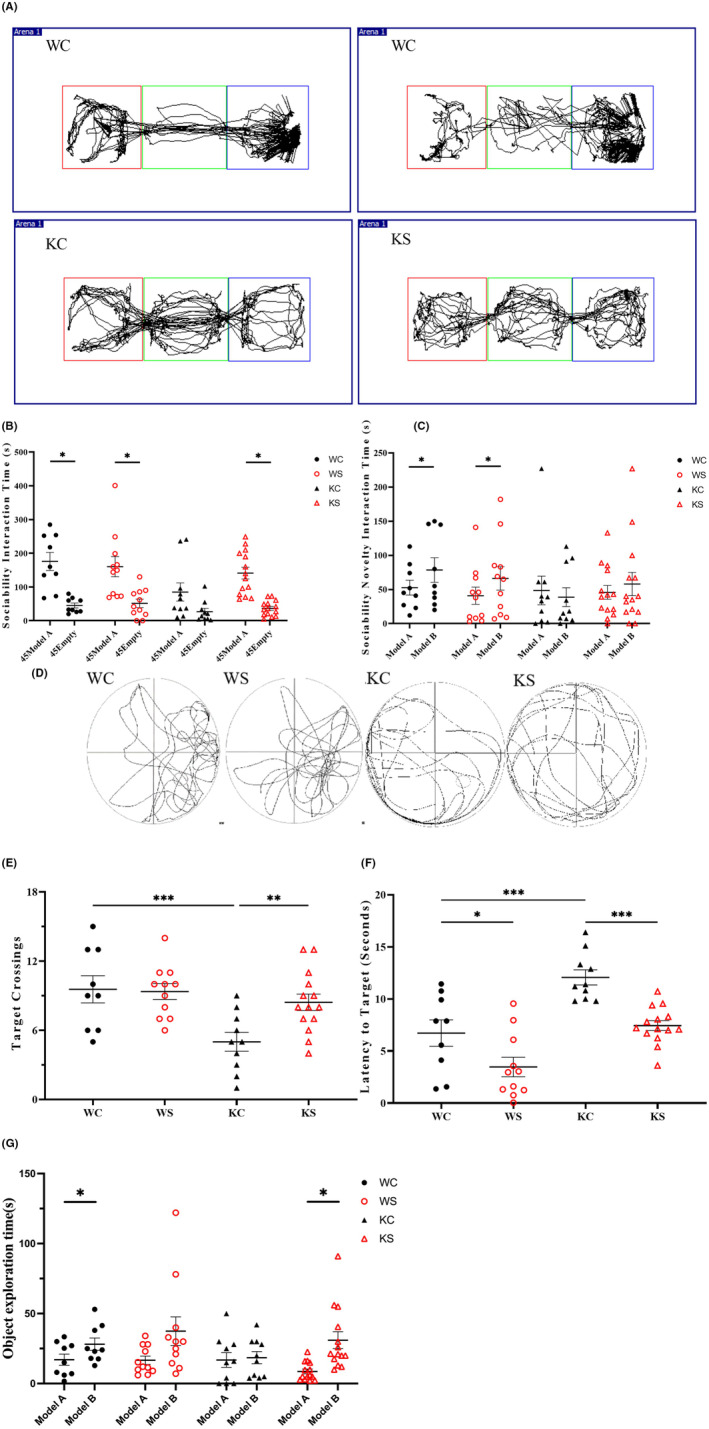
Swimming exercise rescues the learning and memory abilities of Shank 3 KO rats. (A) Influence of 8 weeks of swimming on the social behavior of different groups. Track of social memory in different groups of rats induced by early swimming. (B) The first‐stage social ability results of the three‐chamber test. (C) The second‐stage social memory results of the three‐chamber test. (WC, *n* = 9; WS, *n* = 11; KC, *n* = 10; KS, *n* = 14. Data are presented as the mean ± SEM. *p < 0.05; **p < 0.01; ***p < 0.001). (D) Influence of 8 weeks of swimming on learning and memory of different groups in the water maze. Track chart of the influence of early swimming on water maze learning and the memory of rats in different groups. (E) The number of times that rats have passed through the area where the platform was previously located. (F) The number of times that the rats have passed through the area where the platform was previously located. (G) Influence of 8 weeks of swimming on new and different object recognition in different groups. Data are expressed as the mean ± SEM, ^$^ *p* < 0.05 KC vs. WC, ^$$^ *p* < 0.01 KC vs. WC, *p < 0.05 KC vs. KS, ^#^ *p* < 0.05 WS vs. WC.

As shown in Figure 1A–C, compared with WC rats, KC rats in the first stage of social interaction spent a shorter time in the Model rat cage (Model A: KC, 85.14 ± 8.09 s, WC, 175.89 ± 75.50 s; Empty: KC, 29.52 ± 2.95 s, WC, 45.22 ± 7.30 s). In the second stage, the KC group could not distinguish the new Model B from the old Model A and showed a social memory deficit (KC: Model A, 63.24 ± 6.32 s; Model B, 38.09 ± 8.01 s). There was no significant difference between KS rats and KC rats in the second stage after swimming in the early 8 weeks (*p* > 0.05).

#### 3.1.2 Spatial learning memory

We used the number of target crossings and the latencyto target in morris water maze test to analyze the learning and memory ability. The results showed that the latency to find the platform in KC group was longer compared with that in the WC group (KC: 12.02 ± 0.73 s; WC: 6.72 ± 1.26 s, *p* < 0.0001), and the number of crossings the platform decreased significantly (KC: 5.00 ± 0.82; WC: 9.56 ± 1.18, *p* < 0.0001). Compared with KC, the latency to target was significantly shorter in KS (*p <* 0.0001), and the number of crossings the platform was significantly increased (KS: 8.43 ± 0.71, *p* < 0.001). Compared with WC, the latency to the target time of WS was also significantly reduced (WS: 3.47 ± 0.94, *p <* 0.05), and the number of times crossing the platform was not significantly different (WS: 9.36 ± 0.69, *p >* 0.05). These results indicate that swimming can change the learning and memory ability of KS rats, as shown in Figure 1D–F.

#### 3.1.3 New object memory

New object recognition is often used to evaluate the learning and memory ability of rodents. The results showed that both the WC and WS groups could identify new and old objects. However, KC rats could not distinguish between new and old objects (KC: Novel, 18.49 ± 1.30 s; Familiar, 16.80 ± 1.58 s, *p >* 0.05). In swimming intervention group, the rats could recognize the new and old objects (Novel, 31.00 ± 1.56 s; Familiar, 8.6 ± 0.44 s, *p* < 0.05). Eight weeks of swimming changed the object recognition and memory ability of *Shank3* gene knockout rats, as shown in Figure 1G.

### 3.2 Swimming exercise altered the morphology and structure of Brain of *Shank 3* KO rats

#### 3.2.1 The volume of the hippocampus

All T2‐weighted rats were pretreated and segmented according to the sigma template to complete the pretreatment process. According to the analysis of VBM in small animals, four clusters were found after *p* < 0.01 and FDR correction. Among them, 1192 voxels of VBM were found in the larger cluster, with coordinates of 37.5, −50.6, and −18.15 and a peak intensity of 24.1492. The nuclei involved are the hippocampus, basal ganglia, and other nuclei. The cluster difference test results and the comparison results of the cluster differences are shown in Tables 1 and 2.

**TABLE 1:** Cluster difference test results

Lump name	Voxels
Entorhinal_cotex	634
Cornu_ammonis_1_R	161
Amygdalopiriform_cotex	106
Cornu_ammonis_3_R	99
Subiculum_R	95
Basal_forbrain_region_R	84
Dentate_gyrus_R	10
Lateral_entorhinal_cotex_external_part_R	3
Total	1192

**TABLE 2:** Comparison results of cluster differences

Group	Voxels	*p*
KS vs. KC	15	<0.01
KS vs. WC	956	<0.01
WS vs. WC	25	<0.01
KC vs. WC	Inf	

#### 3.2.2 Lip and (Glu/Gln) in the Hippocampus by ^1^H‐MRS

**FIGURE 2:**
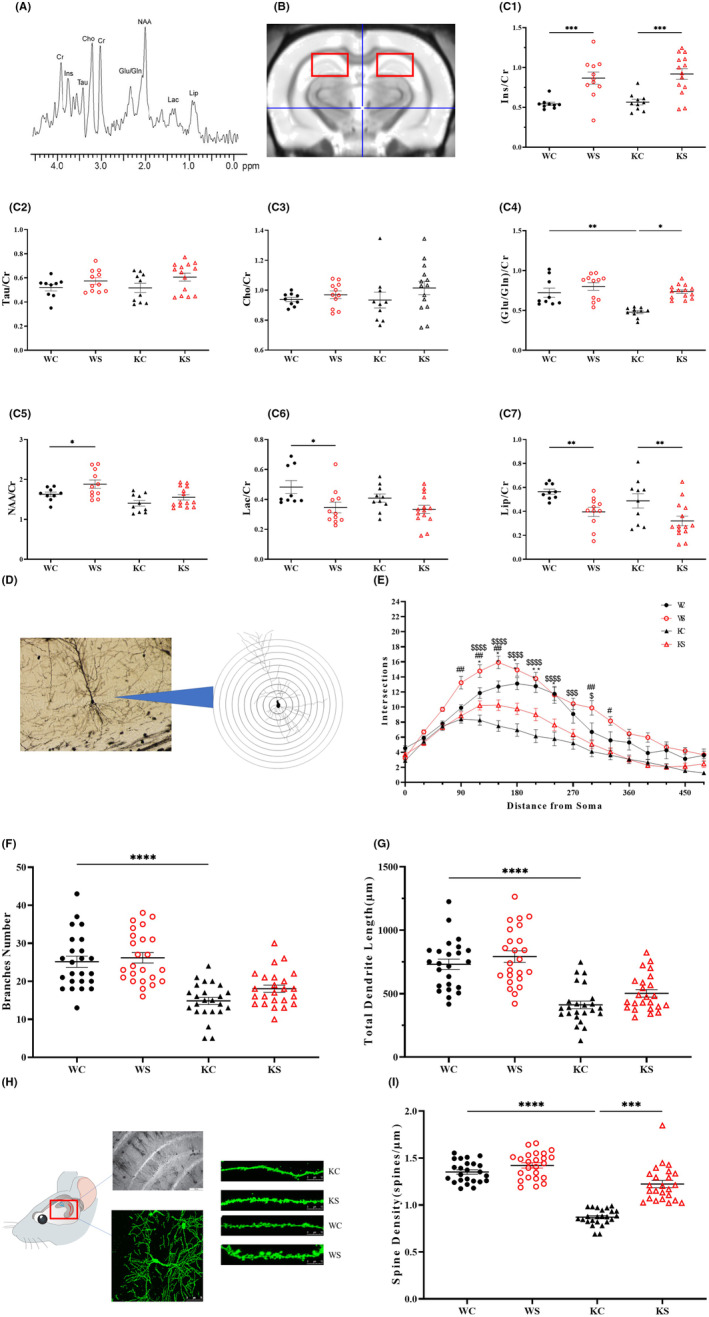
Swimming exercise altered the morphology and structure of Shank 3 KO rats. (A) ^1^H spectrum standard curve. (B) ^1^H spectrum hippocampal compression position schematic. (C) ^1^H‐Pop comparison of the different groups. (D) Electron microscopic observation of neurons in the different groups (100×, standard = 100 μm). The number of secondary branches of hippocampal pyramidal neurons in each group. (E) Average dendrite length. (F) Total branch length. (G) The number of intersections from the cell body. Data are expressed as the mean ± SEM (*n* = 3 per group, each rat had 8 neurons) ^$^ *p* < 0.05 KC vs. WC, ^$$^ *p* < 0.01 KC vs. WC, *p < 0.05 KC vs. KS, ^#^ *p* < 0.05 WS vs. WC. (H) Comparison of the density of dendritic spines in the hippocampus of rats in each group (400×, 10 μm). Representative images of dendritic spines in the hippocampal CA1 neurons. (I) Density of the dendritic spines in the secondary branches of the pyramidal neurons in the hippocampal CA1 region of the rats in each group. Data are expressed as the mean ± SEM (*n* = 3 per group, there were 8 dendritic spines in each group). ^$^ *p* < 0.05 KC vs. WC, *p < 0.05 KC vs. KS.

In this study, the hippocampi of different groups of rats were scanned by ^1^H‐MRS, and nine metabolites were included by LC spectrum analysis. The relative concentration of each metabolite was calculated by the ratio with Cr. The concentration at each time point is shown in Figure 2A,B.

Compared with the WC group, the ratio of (Glu/Gln)/Cr and NAA in the KC group decreased significantly (*p* < 0.001; *p* < 0.05). Compared with the KC group, the ratio of (Glu/Gln)/Cr and Ins/Cr increased in KS group (Figure 2C1–7).

### 3.3 Swimming exercise made hippocampal dendritic branching of neurons more complex in *Shank3* KO rats

#### 3.3.1 The complexity of dendritic branching in the hippocampus

We used electron microscopy to observe the complexity of the dendritic branching in the hippocampus. Under the 10× eyepiece, the morphology of neurons in the CA1 area of the hippocampus in different groups was observed.

As shown in Figure 2D–G, Figure S1C,D the results of Sholl analysis showed that the number of branches in KC was significantly lower than that in WC, and the total branch length decreased significantly. The number of hippocampal dendritic branches was significantly lower in the 120–300 μm segment from the cytosol in the KC group than in the WC group (*p* < 0.001), and the KS group was significantly higher than the KC group in the 120–240 μm segment (*p* < 0.05); the WS group showed a significant increase in the 90–150 μm segment compared with the WC group (*p* < 0.05).

In terms of hippocampal dendritic branch numbers and total dendrite length, the KC group had the shortest total hippocampal dendritic branch length, which was significantly lower than that of the WC group (*p* < 0.0001). In the KS group, although there was some improvement, there was no significant difference compared with the KC group, but the difference was reduced compared with KC vs. WC, KS vs. WC.

#### 3.3.2 Density of dendritic spines in the hippocampus

The dendritic spines of the branches of pyramidal neurons in the hippocampal CA1 region were observed under a laser confocal microscope, and the density of dendritic spines in KCs was significantly lower than that in WCs (*p* < 0.0001). The density of dendritic spines in the KS was significantly higher than that in the KC (*p* < 0.001), and the WS was not significantly different, as shown in Figure 2H,I.

## 4 DISCUSSION

Song et al.^13^ proved that *Shank3* knockdown rats (36–42 days old) had impaired social memory (three‐chamber test) and impaired learning and memory (novel object recognition test). Similarly, we demonstrated that 64‐day *Shank3* knockout rats exhibited hippocampal learning and memory behavior impairment. Then, we performed more extensive behavioral tests of learning memory. We found that the *Shank3* knockout rats had significant deficits in social memory, spatial memory, and object recognition. These behavioral deficits improved after 8 weeks of the swimming intervention. To explain the possible mechanisms by which the swimming intervention improved learning and memory abilities, we examined the morphological structure and proteomics of the hippocampus. The MRI and Golgi staining results indicated that swimming might improve learning memory behavioral performance in rats by modulating the morphological structure of the hippocampus.

In addition to abnormal social and stereotyped behaviors, early motor delay, abnormal gait, and difficulty in gross and fine motor coordination, posture control and imitation are significant neurological comorbidities in the ASD population.^35^, ^36^, ^37^ The quality difference during early exercise behavior may be one of the signs of ASD.^38^, ^39^ In this study, exons 11–21 of the *Shank3* gene were knocked out, and the behavior was observed. In particular, learning and memory disorders were manifested in all of the tests: the three‐chamber test, water maze of spatial learning and memory, and the novel object recognition test.^40^

There have been many reports proving that swimming can improve learning and memory. Our results also found that compare with wild‐type rats, the rats underwent 8 weeks of swimming (WS vs. WC) had less latency to target time (Figure 2F), increased Ins/CR, Tau/CR and reduced Lac/CR,LIP/CR in nuclear magnetic H spectroscopy experiments (Figure 2C1,7), increased distance from soma in neuron Sholl analysis (Figure 2E). These results show that swimming exercise can improve learning and memory, hippocampal neural development, and plasticity in normal rats. These results are also similar to the results of many studies. Alomari et al.^41^ reported that swimming and autonomous running wheel exercise could improve the spatial learning and memory of rats, which may be related to the effect of BDNF on brain plasticity. Wang et al.^42^ showed that 8 weeks of swimming could improve their ability to recognize new and exotic objects and locate them in a water maze through changes in neural plasticity induced by neuronal calcium sensors and calmodulin. Gorantla et al.^43^ also showed that swimming could change the learning and memory deficits of epileptic mice.^43^

However, there are no previous studies on swimming in *Shank3*‐deficient rats. In this study, we found significant improvements in social memory, spatial memory, and object recognition in *Shank3* knockout rats after an 8‐week early swimming intervention, and also, our results found that early swimming did not alter social memory in *Shank3* KO rats, and although swimming greatly altered the structure and morphology of the hippocampus, the brain region that may dominate social memory was not the hippocampus. The literature reports that social interaction in autism is regulated by multiple brain regions, and abnormalities in cortical, and striatal brain regions,^44^ and the literature reports that social interaction in autism is regulated by multiple brain regions, and abnormalities in cortical, and striatal brain regions.^45^

Some studies have shown that an increase in TBV and abnormal white matter is common phenomena in MRI imaging of ASD patients, but there are also changes in the cerebellum, hippocampus, striatum, and thalamus.^46^ Changes in the hippocampal volume in an ASD mouse model are common, and most studies have proven that the hippocampal volume decreases.^47^ In this study, the *Shank3* 11–22 region was knocked out, and no significant volume change was found in the hippocampus. Early swimming intervention increased the volume of the hippocampal gray matter in *Shank3* knockout rats. However, there was no significant difference in the FA and ADC values of the hippocampus in white matter DTI. Jacini et al.^48^ showed that the density of gray matter in judo athletes was significantly higher than that in healthy adults, which reflected the changes in brain plasticity caused by exercise. Cahill et al.^49^ conducted 4 weeks of independent running wheel training on healthy adult mice. MRI tests showed that many brain structures changed, including the hippocampus, cerebral cortex, cerebellum, and other brain areas, and the volume of the hippocampus increased significantly. Using voxel‐based MRI, Biedermann et al.^50^ showed that the volume of the hippocampus in exercised mice increased significantly. The volume of gray matter in the hippocampus in the CA1‐3 region of mice was significantly increased by the use of a self‐running wheel, and the volume of the angular gyrus was increased as shown by VBM analysis.^51^ Running increased and improved the birth of new neurons, the density of dendritic spines, synaptic plasticity, neurotrophin level, and the spatial memory function of mice.^52^ Many animal studies have also used histomorphological techniques to examine the micro‐level changes of the brain structure related to movement and have report an increase in the motor cortex thickness of rats, an increase in the motor cortex neuron number and the cerebellar capillary density in rats, and the relationship between hippocampal cell proliferation and neurogenesis in mice.^53^, ^54^, ^55^, ^56^ These results are consistent with the effect of early swimming intervention on hippocampal formation. However, there was no previous evidence that early swimming can improve *Shank3* knockout rats. Therefore, the mechanism by which swimming exercise increases the volume of hippocampal gray matter in *Shank3* knockout rats needs further study.

The involvement of AMPAR, NMDAR, and mGluR is required for the delivery of glutamate (Glu) excitatory signals, and multiple studies have confirmed that the *Shank3* gene can affect these 3 classes of receptors and hence the transmission of neural signaling.^57^ They are considered potential therapeutic targets for ASD diseases.^58^ Some studies have pointed out that Glu and Gln in children with ASD can be used as biomarkers for an early diagnosis. The serum level of Glu in young people with ASD is significantly higher than that in the adult control group.^59^, ^60^ Fatemi et al.^61^ demonstrated that in the brains of ASD patients, the levels of GAD 65 kDa and GAD 67 kDa proteins (both involved in the conversion of Glu to GABA) decreased, resulting in increased Glu levels in the brain. Glutathione increases in hippocampal CA3 neurons of *Shank3* knockout mice during early development.^62^ The MRS results showed that *Shank3* gene knockout reduced Glu/Gln in the hippocampus of rats, which was consistent with some previous studies, but they did not show differences in other metabolites. This may be related to the specific knockout fragment in this study, which needs further study. MRS measurement after yoga training for 12 weeks showed that the concentration of GABA in the hippocampus increased, which may be related to the lower anxiety induced by yoga.^63^ Maddock et al.^64^ showed that after extreme exercise, ^1^H‐MRS in the brain showed an increase in lactate signaling. This study also showed that early swimming can increase Glu/Gln, decrease Lip, and increase Ins in KO rats. The significant changes in the gray matter volume and metabolites indicate that the hippocampus is the core brain area affected by early swimming intervention. *Shank3* gene knockout rats were subjected to early swimming intervention, and the structural plasticity of the hippocampus can be further explored by cell tissue technology.

Many studies have shown that morphological changes in dendritic spines are related to the pathogenesis of ASD.^65^ During the process of synaptic plasticity, the size and shape of dendritic spines will change. This process constantly reshapes the brain circuits in response to external stimuli and induces the formation of learning and memory.^66^ At present, a variety of animal models with gene defects have been constructed. Dendritic spine deletion has been found in several ASD animal models, such as TSC1 mutant mice,^67^ UBE3A mutant mice,^68^ MeCP2 knockout mice,^69^ cntnap2 knockout mice,^70^ and VPA‐exposed rats and mice.^71^, ^72^ In ASD patients, *Shank3* gene defects have been observed. It encodes a synaptic scaffold protein involved in the induction and maintenance of dendritic spines and it regulates the shape and density of dendritic spines.^73^, ^74^ In addition, previous studies have shown that exercise intervention can change synaptic plasticity by increasing dendritic spines. Exercise regulates dendritic spine plasticity by activating BDNF‐ and IGF‐mediated intracellular signaling pathways, including protein kinases (MAPK/ERK and CAM‐k) and endothelial nitric oxide synthase (eNOS).^75^ Exercise may regulate the morphology and number of dendritic spines by changing the postsynaptic receptor proteins and the cellular molecular mechanisms. *Shank3* gene knockout rats have a decreased density of dendritic spines. Therefore, the process of increasing dendritic spines during early swimming may be caused by changes in postsynaptic receptor proteins and cellular molecular mechanisms, but the mechanism needs to be explored further.

In conclusion, this study demonstrated that 8 weeks of swimming is effective in improving the behavioral phenotype related to autism comorbidity and promotes the growth of neurons and the expression of related proteins in *Shank3*‐deficient rats. This confirms the potential of swimming for the treatment of autism and other neurodevelopmental disorders and provides positive evidence for clinical practice. It is worth noting that further experimental verification may be needed to understand the specific mechanism by which swimming improves autism.

## FUNDING INFORMATION

This study was supported by the Key Scientific and Technological projects of Guangdong Province (2018B30335001), the Key Project of Beijing Social Science Foundation (No.19YTA007), the National Basic Research Program of China (2017YFA0105201) and Beijing Municipal Science & Technology Commission (Z181100001518005).

## CONFLICT OF INTEREST

The authors declare that they have no conflict of interest.

## Supporting information


Figure S1

## Data Availability

The data that support the findings of this study are available from the corresponding author upon reasonable request. Some data that supports the findings of this study are available in the Supplementary Material of this article.

## References

[1] Maenner MJ , Shaw KA , Bakian AV , et al. Prevalence and characteristics of autism spectrum disorder among children aged 8 years – autism and developmental disabilities monitoring network, 11 sites, United States, 2018. MMWR Surveill Summ. 2021;70(11):1‐16. doi:10.15585/mmwr.ss7011a1 PMC8639024 34855725

[2] Delling JP , Boeckers TM . Comparison of SHANK3 deficiency in animal models: phenotypes, treatment strategies, and translational implications. J Neurodev Disord. 2021;13(1):55. doi:10.1186/s11689-021-09397-8 34784886 PMC8594088

[3] Cheroni C , Caporale N , Testa G . Autism spectrum disorder at the crossroad between genes and environment: contributions, convergences, and interactions in ASD developmental pathophysiology. Mol Autism. 2020;11(1):69. doi:10.1186/s13229-020-00370-1 32912338 PMC7488083

[4] Trambacz‐Oleszak S , Nosulia T . Genetic factors in autism spectrum disorders (ASD). Postepy Biochem. 2021;67(1):28‐33. doi:10.18388/pb.2021_377 34378896

[5] Barak B , Feng GP . Neurobiology of social behavior abnormalities in autism and Williams syndrome. Nat Neurosci. 2016;19(5):647‐655. doi:10.1038/nn.4276 29323671 PMC4896837

[6] Boccuto L , Lauri M , Sarasua SM , et al. Prevalence of SHANK3 variants in patients with different subtypes of autism spectrum disorders. Eur J Hum Genet. 2013;21(3):310‐316. doi:10.1038/ejhg.2012.175 22892527 PMC3573207

[7] Durand CM , Betancur C , Boeckers TM , et al. Mutations in the gene encoding the synaptic scaffolding protein SHANK3 are associated with autism spectrum disorders. Nat Genet. 2007;39(1):25‐27. doi:10.1038/ng1933 17173049 PMC2082049

[8] Bourgeron T . From the genetic architecture to synaptic plasticity in autism spectrum disorder. Nat Rev Neurosci. 2015;16(9):551‐563. doi:10.1038/nrn3992 26289574

[9] Gilman SR , Iossifov I , Levy D , Ronemus M , Wigler M , Vitkup D . Rare de novo variants associated with autism implicate a large functional network of genes involved in formation and function of synapses. Neuron. 2011;70(5):898‐907. doi:10.1016/j.neuron.2011.05.021 21658583 PMC3607702

[10] Sharma SR , Gonda X , Tarazi FI . Autism spectrum disorder: classification, diagnosis and therapy. Pharmacol Ther. 2018;190:91‐104. doi:10.1016/j.pharmthera.2018.05.007 29763648

[11] Du YR , Weed SA , Xiong WC , Marshall TD , Parsons JT . Identification of a novel cortactin SH3 domain‐binding protein and its localization to growth cones of cultured neurons. Mol Cell Biol. 1998;18(10):5838‐5851. doi:10.1128/Mcb.18.10.5838 9742101 PMC109170

[12] Lee EJ , Choi SY , Kim E . NMDA receptor dysfunction in autism spectrum disorders. Curr Opin Pharmacol. 2015;20:8‐13. doi:10.1016/j.coph.2014.10.007 25636159

[13] Song TJ , Lan XY , Wei MP , et al. Altered behaviors and impaired synaptic function in a novel rat model with a complete Shank3 deletion. Front Cell Neurosci. 2019;13:111. doi:10.3389/fncel.2019.00111 30971895 PMC6444209

[14] Tu Z , Zhao H , Li B , et al. CRISPR/Cas9‐mediated disruption of SHANK3 in monkey leads to drug‐treatable autism‐like symptoms. Hum Mol Genet. 2019;28(4):561‐571. doi:10.1093/hmg/ddy367 30329048 PMC6489410

[15] Wu S , Gan G , Zhang Z , et al. A presynaptic function of shank protein in drosophila. J Neurosci. 2017;37(48):11592‐11604. doi:10.1523/JNEUROSCI.0893-17.2017 29074576 PMC6705749

[16] Zhao H , Tu Z , Xu H , et al. Altered neurogenesis and disrupted expression of synaptic proteins in prefrontal cortex of SHANK3‐deficient non‐human primate. Cell Res. 2017;27(10):1293‐1297. doi:10.1038/cr.2017.95 28741620 PMC5630686

[17] Zhou Y , Sharma J , Ke Q , et al. Atypical behaviour and connectivity in SHANK3‐mutant macaques. Nature. 2019;570(7761):326‐331. doi:10.1038/s41586-019-1278-0 31189958

[18] Chen YC , Baram TZ . Toward understanding how early‐life stress reprograms cognitive and emotional brain networks. Neuropsychopharmacology. 2016;41(1):197‐206. doi:10.1038/npp.2015.181 26105143 PMC4677123

[19] Matsuda F , Sakakima H , Yoshida Y . The effects of early exercise on brain damage and recovery after focal cerebral infarction in rats. Acta Physiol. 2011;201(2):275‐287. doi:10.1111/j.1748-1716.2010.02174.x PMC3045711 20726846

[20] Zhang PY , Zhang YL , Zhang J , et al. Early exercise protects against cerebral ischemic injury through inhibiting neuron apoptosis in cortex in rats. Int J Mol Sci. 2013;14(3):6074‐6089. doi:10.3390/ijms14036074 23502470 PMC3634421

[21] Grzadzinski R , Amso D , Landa R , et al. Pre‐symptomatic intervention for autism spectrum disorder (ASD): defining a research agenda. J Neurodev Disord. 2021;13(1):49. doi:10.1186/s11689-021-09393-y 34654371 PMC8520312

[22] Myers AJ , Cleveland E , Whitby PJS , et al. Analysis of a statewide early intervention program for young children with ASD. J Autism Dev Disord. 2021:1‐13. doi:10.1007/s10803-021-05376-z 34797471

[23] Thomas S , Hinkley T , Barnett LM , May T , Rinehart N . Young children with ASD participate in the same level of physical activity as children without ASD: implications for early intervention to maintain good health. J Autism Dev Disord. 2019;49(8):3278‐3289. doi:10.1007/s10803-019-04026-9 31079278

[24] Da Silva LA , Doyenart R , Salvan PH , et al. Swimming training improves mental health parameters, cognition and motor coordination in children with attention deficit hyperactivity disorder. Int J Environ Health Res. 2020;30(5):584‐592. doi:10.1080/09603123.2019.1612041 31081373

[25] Hillman CH , Castelli DM , Buck SM . Aerobic fitness and neurocognitive function in healthy preadolescent children. Med Sci Sports Exerc. 2005;37(11):1967‐1974. doi:10.1249/01.mss.0000176680.79702.ce 16286868

[26] Caputo G , Ippolito G , Mazzotta M , et al. Effectiveness of a multisystem aquatic therapy for children with autism spectrum disorders. J Autism Dev Disord. 2018;48(6):1945‐1956. doi:10.1007/s10803-017-3456-y 29313176

[27] Fragala‐Pinkham MA , Haley SM , O'Neil ME . Group swimming and aquatic exercise programme for children with autism spectrum disorders: a pilot study. Dev Neurorehabil. 2011;14(4):230‐241. doi:10.3109/17518423.2011.575438 21732807

[28] Mische Lawson L , D'Adamo J , Campbell K , et al. A qualitative investigation of swimming experiences of children with autism spectrum disorders and their families. Clin Med Insights Pediatr. 2019;13:1179556519872214. doi:10.1177/1179556519872214 35153525 PMC8826265

[29] Chu CH , Pan CY . The effect of peer‐ and sibling‐assisted aquatic program on interaction behaviors and aquatic skills of children with autism spectrum disorders and their peers/siblings. Res Autism Spectr Disord. 2012;6(3):1211‐1223. doi:10.1016/j.rasd.2012.02.003

[30] Alaniz ML , Rosenberg SS , Beard NR , Rosario ER . The effectiveness of aquatic group therapy for improving water safety and social interactions in children with autism spectrum disorder: a pilot program. J Autism Dev Disord. 2017;47(12):4006‐4017. doi:10.1007/s10803-017-3264-4 28864911

[31] Zanobini M , Solari S . Effectiveness of the program "Acqua Mediatrice di Comunicazione" (Water as a Mediator of Communication) on social skills, autistic behaviors and aquatic skills in ASD children. J Autism Dev Disord. 2019;49(10):4134‐4146. doi:10.1007/s10803-019-04128-4 31267291

[32] Andreollo NA , Santos EF , Araujo MR , Lopes LR . Rat's age versus human's age: what is the relationship? Arq Bras Cir Dig. 2012;25(1):49‐51. doi:10.1590/s0102-67202012000100011 22569979

[33] Silva‐Gondim MBE , de Souza TKM , Rodrigues MCA , Guedes RCA . Suckling in litters with different sizes, and early and late swimming exercise differentially modulates anxiety‐like behavior, memory and electrocorticogram potentiation after spreading depression in rats. Nutr Neurosci. 2019;22(7):464‐473. doi:10.1080/1028415x.2017.1407472 29183255

[34] Jorgensen BP , Krych L , Pedersen TB , et al. Investigating the long‐term effect of subchronic phencyclidine‐treatment on novel object recognition and the association between the gut microbiota and behavior in the animal model of schizophrenia. Physiol Behav. 2015;141:32‐39. doi:10.1016/j.physbeh.2014.12.042 25545766

[35] Bhat AN , Landa RJ , Galloway JC . Current perspectives on motor functioning in infants, children, and adults with autism spectrum disorders. Phys Ther. 2011;91(7):1116‐1129. doi:10.2522/ptj.20100294 21546566

[36] Maski KP , Jeste SS , Spence SJ . Common neurological co‐morbidities in autism spectrum disorders. Curr Opin Pediatr. 2011;23(6):609‐615. doi:10.1097/MOP.0b013e32834c9282 21970828 PMC4229811

[37] McCleery JP , Elliott NA , Sampanis DS , Stefanidou CA . Motor development and motor resonance difficulties in autism: relevance to early intervention for language and communication skills. Front Integr Neurosci. 2013;7:30. doi:10.3389/fnint.2013.00030 23630476 PMC3634796

[38] Chawarska K , Volkmar F . Impairments in monkey and human face recognition in 2‐year‐old toddlers with autism spectrum disorder and developmental Delay. Dev Sci. 2007;10(2):266‐279. doi:10.1111/j.1467-7687.2006.00543.x 17286849

[39] Ketcheson L , Hauck J , Ulrich D . The effects of an early motor skill intervention on motor skills, levels of physical activity, and socialization in young children with autism spectrum disorder: a pilot study. Autism. 2017;21(4):481‐492. doi:10.1177/1362361316650611 27354429

[40] Lee J , Chung C , Ha S , et al. Shank3‐mutant mice lacking exon 9 show altered excitation/inhibition balance, enhanced rearing, and spatial memory deficit. Front Cell Neurosci. 2015;9:94. doi:10.3389/fncel.2015.00094 25852484 PMC4365696

[41] Alomari MA , Khabour OF , Alzoubi KH , Alzubi MA . Forced and voluntary exercises equally improve spatial learning and memory and hippocampal BDNF levels. Behav Brain Res. 2013;247:34‐39. doi:10.1016/j.bbr.2013.03.007 23499703

[42] Wang D , Li B , Wu Y , Li B . The effects of maternal atrazine exposure and swimming training on spatial learning memory and hippocampal morphology in offspring male rats via PSD95/NR2B signaling pathway. Cell Mol Neurobiol. 2019;39(7):1003‐1015. doi:10.1007/s10571-019-00695-3 31187311 PMC11457838

[43] Gorantla VR . Effects of swimming exercise on learning and memory in the kainate‐lesion model of temporal lobe epilepsy. J Clin Diagn Res. 2016;10:CF01‐CF05. doi:10.7860/jcdr/2016/22100.8835 PMC5198314 28050361

[44] Bey AL , Wang XM , Yan HD , et al. Brain region‐specific disruption of Shank3 in mice reveals a dissociation for cortical and striatal circuits in autism‐related behaviors. Transl Psychiatry. 2018;8:94. doi:10.1038/s41398-018-0142-6 29700290 PMC5919902

[45] Guo BL , Chen J , Chen Q , et al. Anterior cingulate cortex dysfunction underlies social deficits in Shank3 mutant mice. Nat Neurosci. 2019;22(8):1223‐1234. doi:10.1038/s41593-019-0445-9 31332372

[46] Stigler KA , McDonald BC , Anand A , Saykin AJ , McDougle CJ . Structural and functional magnetic resonance imaging of autism spectrum disorders. Brain Res. 2011;1380:146‐161. doi:10.1016/j.brainres.2010.11.076 21130750 PMC3465665

[47] Ellegood J , Lerch JP , Henkelman RM . Brain abnormalities in a neuroligin3 R451C knockin mouse model associated with autism. Autism Res. 2011;4(5):368‐376. doi:10.1002/aur.215 21882360

[48] Jacini WF , Cannonieri GC , Fernandes PT , Bonilha L , Cendes F , Li LM . Can exercise shape your brain? Cortical differences associated with judo practice. J Sci Med Sport. 2009;12(6):688‐690. 19147406 10.1016/j.jsams.2008.11.004

[49] Cahill LS , Steadman PE , Jones CE , et al. MRI‐detectable changes in mouse brain structure induced by voluntary exercise. NeuroImage. 2015;113:175‐183. doi:10.1016/j.neuroimage.2015.03.036 25800209

[50] Biedermann S , Fuss J , Zheng L , et al. In vivo voxel based morphometry: detection of increased hippocampal volume and decreased glutamate levels in exercising mice. NeuroImage. 2012;61(4):1206‐1212. doi:10.1016/j.neuroimage.2012.04.010 22521257

[51] Sack M , Lenz JN , Jakovcevski M , et al. Early effects of a high‐caloric diet and physical exercise on brain volumetry and behavior: a combined MRI and histology study in mice. Brain Imaging Behav. 2017;11(5):1385‐1396. doi:10.1007/s11682-016-9638-y 27734300 PMC5653704

[52] Lezi E , Lu J , Burns JM , Swerdlow RH . Effect of exercise on mouse liver and brain bioenergetic infrastructures. Exp Physiol. 2013;98(1):207‐219. doi:10.1113/expphysiol.2012.066688 22613742 PMC3540163

[53] Anderson MF , Sims NR . The effects of focal ischemia and reperfusion on the glutathione content of mitochondria from rat brain subregions. J Neurochem. 2002;81(3):541‐549. doi:10.1046/j.1471-4159.2002.00836.x 12065662

[54] Isaacs KL , Sartor RB , Haskill S . Cytokine messenger RNA profiles in inflammatory bowel disease mucosa detected by polymerase chain reaction amplification. Gastroenterology. 1992;103(5):1587‐1595. doi:10.1016/0016-5085(92)91182-4 1426879

[55] Kleim JA , Barbay S , Cooper NR , et al. Motor learning‐dependent synaptogenesis is localized to functionally reorganized motor cortex. Neurobiol Learn Mem. 2002;77(1):63‐77. doi:10.1006/nlme.2000.4004 11749086

[56] van Praag H , Christie BR , Sejnowski TJ , Gage FH . Running enhances neurogenesis, learning, and long‐term potentiation in mice. Proc Natl Acad Sci U S A. 1999;96(23):13427‐13431. doi:10.1073/pnas.96.23.13427 10557337 PMC23964

[57] Monteiro P , Feng GP . SHANK proteins: roles at the synapse and in autism spectrum disorder. Nat Rev Neurosci. 2017;18(3):147‐157. doi:10.1038/nrn.2016.183 28179641

[58] Choudhury PR , Lahiri S , Rajamma U . Glutamate mediated signaling in the pathophysiology of autism spectrum disorders. Pharmacol Biochem Behav. 2012;100(4):841‐849. doi:10.1016/j.pbb.2011.06.023 21756930

[59] Shimmura C , Suda S , Tsuchiya KJ , et al. Alteration of plasma glutamate and glutamine levels in children with high‐functioning autism. PLoS One. 2011;6(10):e25340. doi:10.1371/journal.pone.0025340 21998651 PMC3187770

[60] Shinohe A , Hashimoto K , Nakamura K , et al. Increased serum levels of glutamate in adult patients with autism. Prog Neuro‐Psychopharmacol Biol Psychiatry. 2006;30(8):1472‐1477. doi:10.1016/j.pnpbp.2006.06.013 16863675

[61] Fatemi SH , Halt AR , Stary JM , Kanodia R , Schulz SC , Realmuto GR . Glutamic acid decarboxylase 65 and 67 kDa proteins are reduced in autistic parietal and cerebellar cortices. Biol Psychiatry. 2002;52(8):805‐810. doi:10.1016/s0006-3223(02)01430-0 12372652

[62] Chiesa M , Nardou R , Lozovaya N , et al. Enhanced glutamatergic currents at birth in Shank3 KO mice. Neural Plast. 2019;2019:2382639. doi:10.1155/2019/2382639 31354805 PMC6636579

[63] Streeter CC , Whitfield TH , Owen L , et al. Effects of yoga versus walking on mood, anxiety, and brain GABA levels: a randomized controlled MRS study. J Altern Complement Med. 2010;16(11):1145‐1152. doi:10.1089/acm.2010.0007 20722471 PMC3111147

[64] Maddock RJ , Casazza GA , Buonocore MH , Tanase C . Vigorous exercise increases brain lactate and Glx (glutamate plus glutamine): a dynamic 1H‐MRS study. NeuroImage. 2011;57(4):1324‐1330. doi:10.1016/j.neuroimage.2011.05.048 21640838

[65] Wang Y , Wang W , Wang L , Wang J , Tang J . Ultrasound‐guided high‐intensity focused ultrasound treatment for abdominal wall endometriosis: preliminary results. Eur J Radiol. 2011;79(1):56‐59. doi:10.1016/j.ejrad.2009.12.034 20116953

[66] Menna E , Zambetti S , Morini R , et al. Eps8 controls dendritic spine density and synaptic plasticity through its actin‐capping activity. EMBO J. 2013;32(12):1730‐1744. doi:10.1038/emboj.2013.107 23685357 PMC3680733

[67] Meikle L , Pollizzi K , Egnor A , et al. Response of a neuronal model of tuberous sclerosis to mammalian target of rapamycin (mTOR) inhibitors: effects on mTORC1 and Akt signaling lead to improved survival and function. J Neurosci. 2008;28(21):5422‐5432. doi:10.1523/jneurosci.0955-08.2008 18495876 PMC2633923

[68] Dindot SV , Antalffy BA , Bhattacharjee MB , Beaudet AL . The Angelman syndrome ubiquitin ligase localizes to the synapse and nucleus, and maternal deficiency results in abnormal dendritic spine morphology. Hum Mol Genet. 2008;17(1):111‐118. doi:10.1093/hmg/ddm288 17940072

[69] Asaka Y , Jugloff DGM , Zhang LA , Eubanks JH , Fitzsimonds RM . Hippocampal synaptic plasticity is impaired in the Mecp2‐null mouse model of Rett syndrome. Neurobiol Dis. 2006;21(1):217‐227. doi:10.1016/j.nbd.2005.07.005 16087343

[70] Gdalyahu A , Lazaro M , Penagarikano O , Golshani P , Trachtenberg JT , Gescwind DH . The autism related protein contactin‐associated protein‐like 2 (CNTNAP2) stabilizes new spines: an in vivo mouse study. PLoS One. 2015;10(5):e0125633. doi:10.1371/journal.pone.0125633 25951243 PMC4423902

[71] Hou Q , Wang Y , Li Y , Chen D , Yang F , Wang S . A developmental study of abnormal behaviors and altered GABAergic signaling in the VPA‐treated rat model of autism. Front Behav Neurosci. 2018;12:182. doi:10.3389/fnbeh.2018.00182 30186123 PMC6110947

[72] Wang R , Tan J , Guo J , et al. Aberrant development and synaptic transmission of cerebellar cortex in a VPA induced mouse autism model. Front Cell Neurosci. 2018;12:500. doi:10.3389/fncel.2018.00500 30622458 PMC6308145

[73] Boeckers TM , Bockmann J , Kreutz MR , Gundelfinger ED . ProSAP/Shank proteins – a family of higher order organizing molecules of the postsynaptic density with an emerging role in human neurological disease. J Neurochem. 2002;81(5):903‐910. doi:10.1046/j.1471-4159.2002.00931.x 12065602

[74] Hotulainen P , Hoogenraad CC . Actin in dendritic spines: connecting dynamics to function. J Cell Biol. 2010;189(4):619‐629. doi:10.1083/jcb.201003008 20457765 PMC2872912

[75] Vaynman S , Ying Z , Gomez‐Pinilla F . Interplay between brain‐derived neurotrophic factor and signal transduction modulators in the regulation of the effects of exercise on synaptic‐plasticity. Neuroscience. 2003;122(3):647‐657. doi:10.1016/j.neuroscience.2003.08.001 14622908

