# Supplementary figures and images for: Swimming exercise is a promising early intervention for autism‐like behavior in Shank3 deletion rats
Source: CNS Neurosci Ther. 2022 Oct 11;29(1):78–90. doi: 10.1111/cns.13920 (PMC9804047; doi:10.1111/cns.13920)

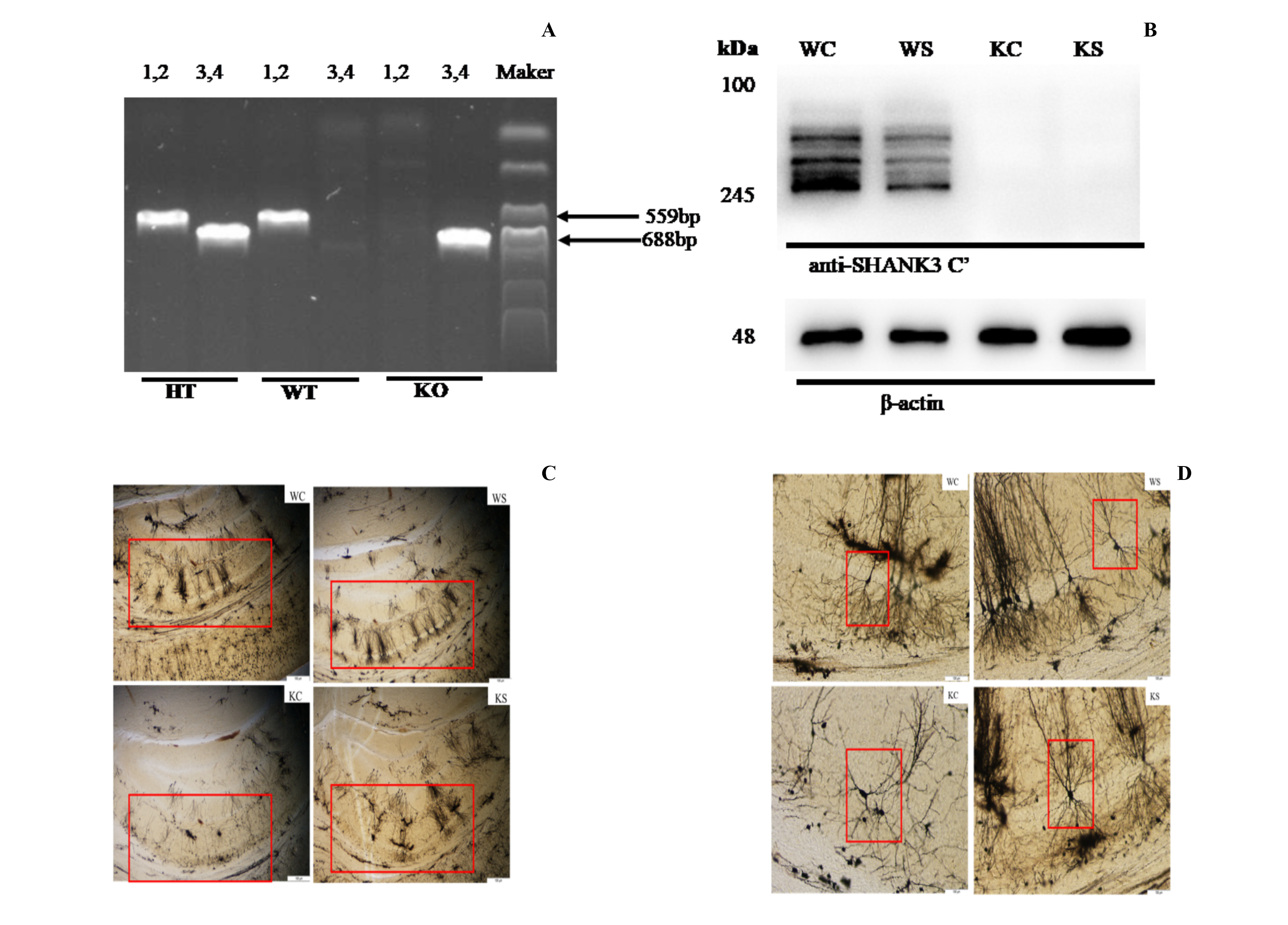

Supplement: Supplementary file 1 — Figure S1 [file CNS-29-78-s001.tif]
